# Adult intussusception secondary to an appendiceal tumour in a patient with ulcerative colitis: a case report

**DOI:** 10.1186/s40792-020-01017-2

**Published:** 2020-09-29

**Authors:** M. G. Davey, E. T. Conlon, G. Forde, V. M. Byrnes, P. A. Carroll

**Affiliations:** 1 Department of Surgery, Galway University Hospitals, Galway, Ireland; 2 School of Medicine and Health Sciences, University College Dublin, Dublin 4, Ireland; 3 Department of Gastroenterology and Hepatology, Galway University Hospitals, Galway, Ireland

**Keywords:** LAMN, Intussusception, IBD, Ulcerative colitis

## Abstract

**Background:**

Intussusception in adult patients is uncommon and appendiceal lead points are particularly rare.

**Case presentation:**

We present the case of a 42-year-old male with a history of ulcerative colitis, presenting with sudden onset abdominal pain and bloody diarrhoea. Endoscopy revealed grossly normal mucosa in the descending colon with a congested polypoid mass in the proximal transverse colon. Computed tomography revealed ileocecal intussusception at the hepatic flexure. A right hemicolectomy was performed, where a grossly dilated appendix was noted, resected and sent for histopathological evaluation. Results revealed low-grade appendiceal mucinous neoplasm. Post-operatively, the patient remained symptom free, however required reintroduction of biologic therapy due to relapse of his ulcerative colitis 12 weeks later.

**Conclusion:**

This case depicts a rare acute surgical presentation and reminds physicians and surgeons of the importance of ‘thinking outside the box’ in clinical practice.

## Introduction

Ulcerative colitis (UC) is the most common form of inflammatory bowel disease (IBD), with an incidence of approximately 2 million in European populations [1]. UC is a multifactorial inflammatory condition, where interplay of inherited and environmental factors cause inappropriate immune responses of a remitting and relapsing nature [2]. Adult intussusception is a rare presentation and the estimated incidence of appendiceal intussusception is 0.01% [3]. Adult intussusception typically requires surgical intervention on account of the high incidence of occurrence within the setting of neoplasms [4]. Herein, we present a complex case of a male patient presenting to the emergency department of University Teaching Hospital with a perceived exacerbation of UC.

## Case report

**Fig. 1:**
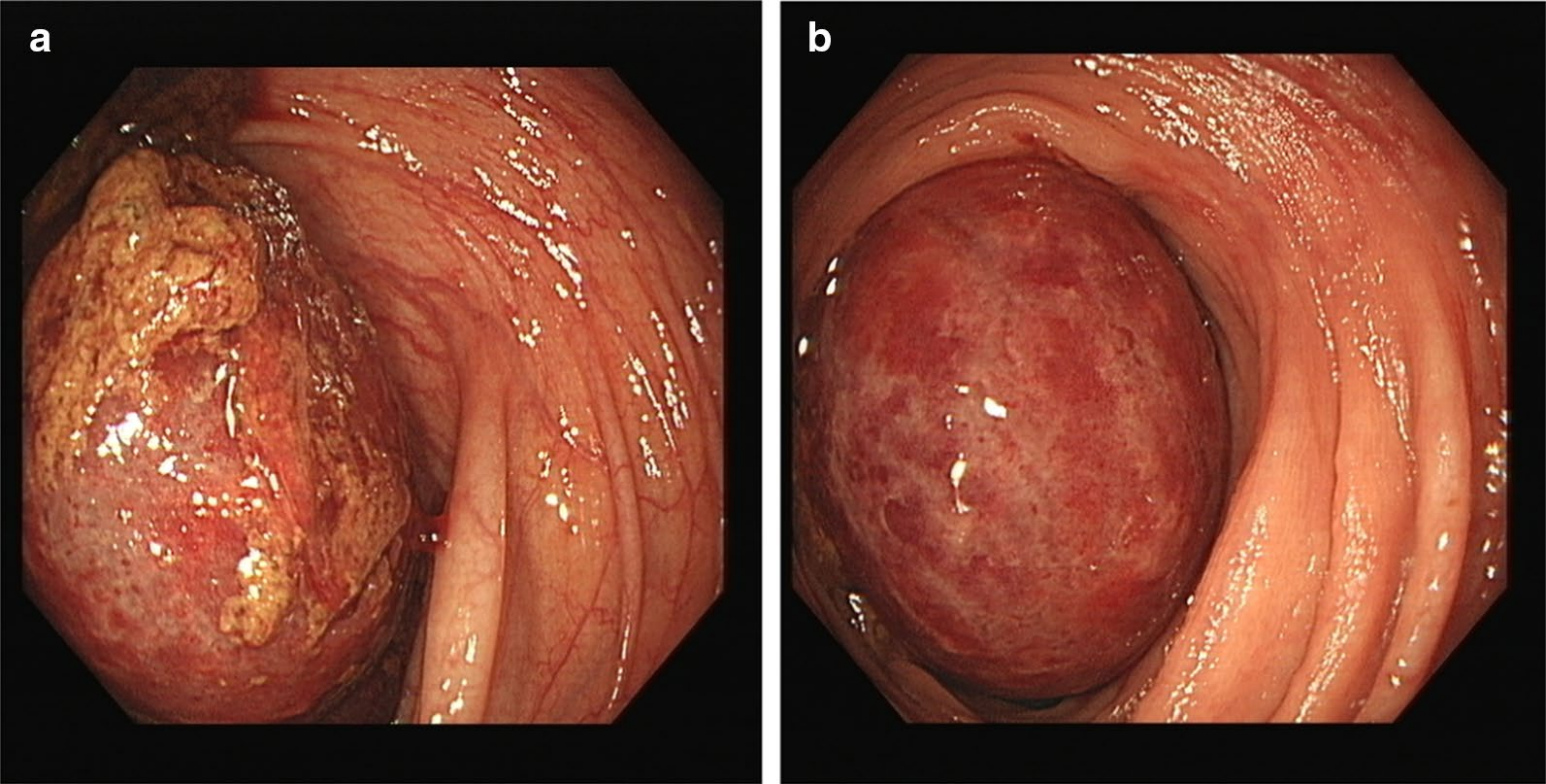
Endoscopic images captured during flexible sigmoidoscopy; obvious intramural globular mass detected at the proximal aspect of the transverse colon

**Fig. 2:**
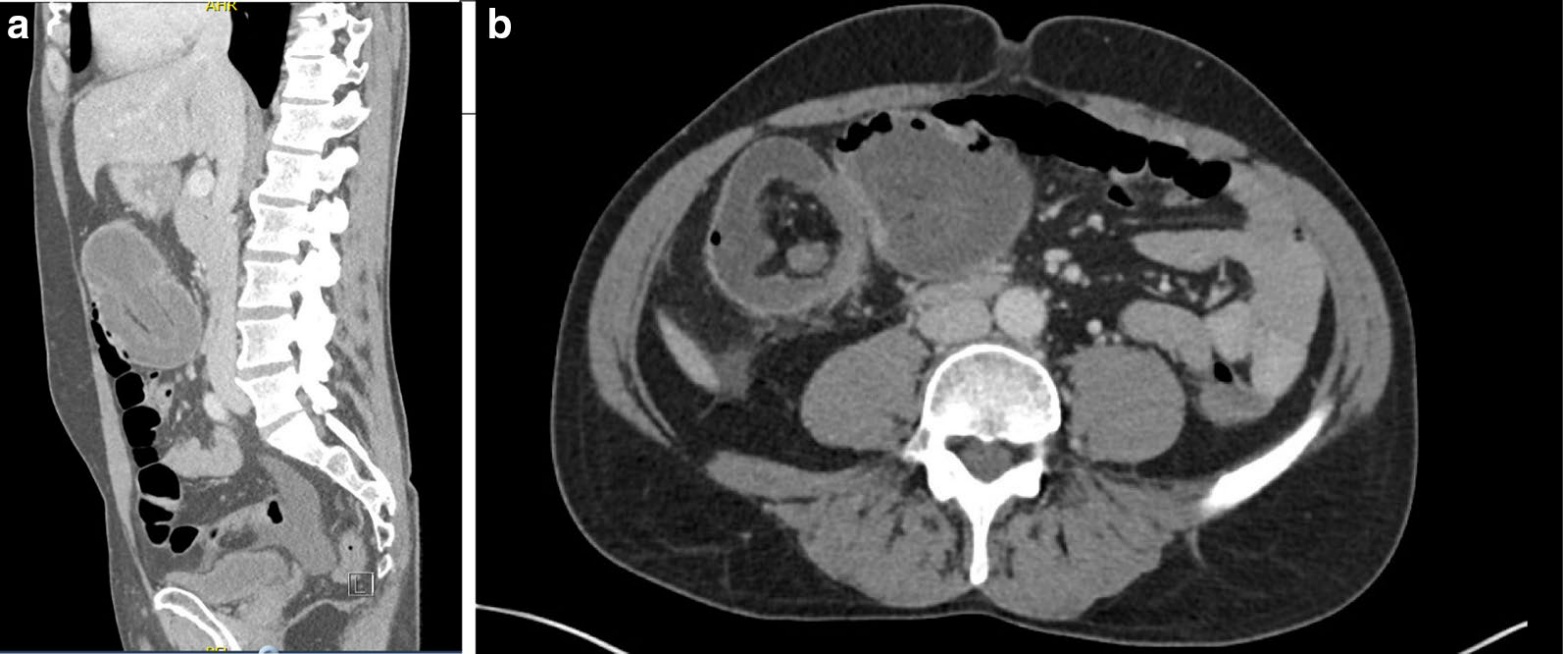
Commuted tomographic abdominal pelvic imaging of **a** sagittal, and **b** coronal planes demonstrating ileocaecal intussusception at the hepatic flexure. No obvious lead point was detected on radiological imaging

**Fig. 3:**
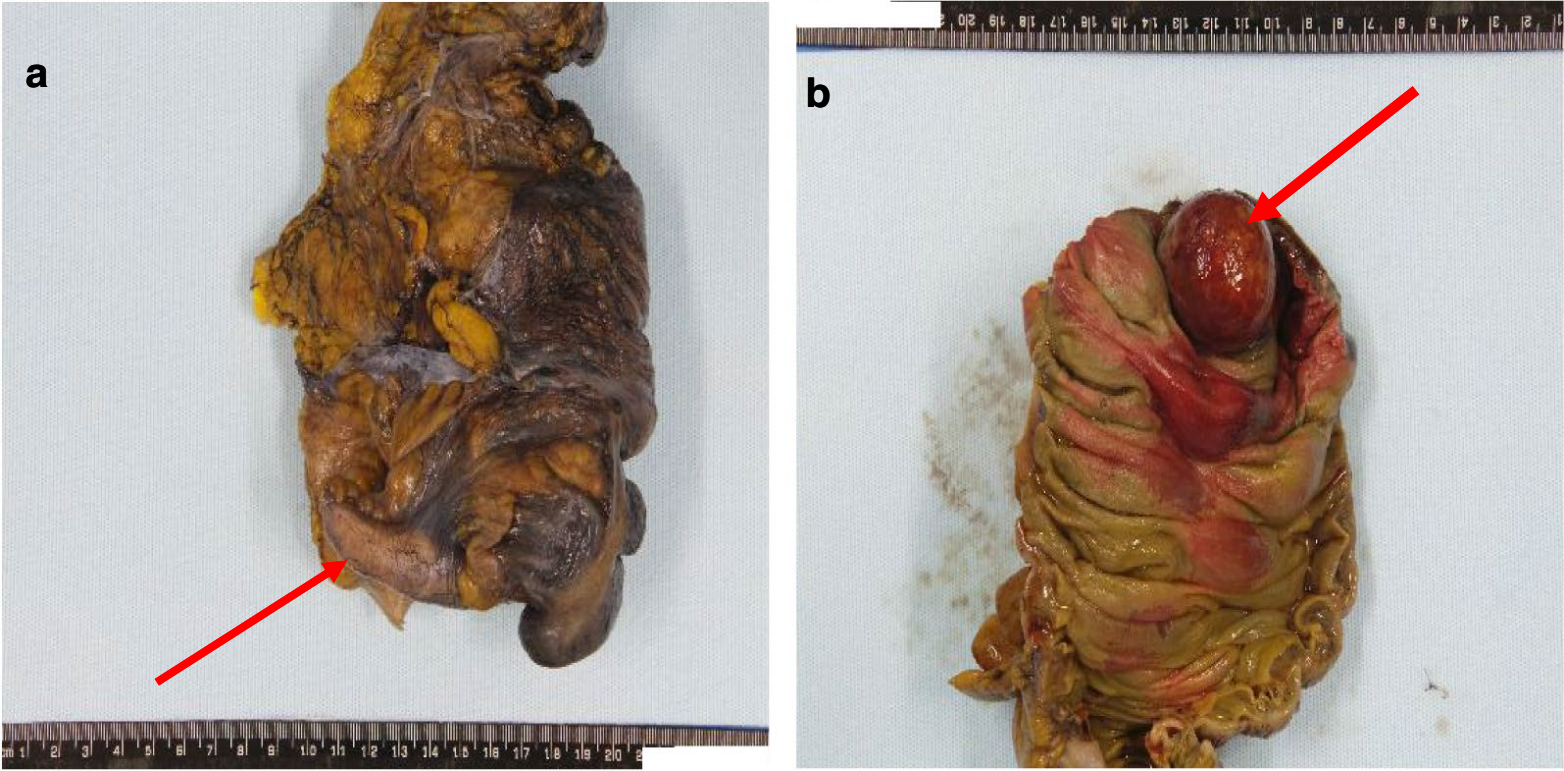
Macroscopic images of resected specimens: **a** gross appendix externally at caecal pole, **b** extruding mass into caecal lumen from the appendix. Histopathological evaluation revealed *‘vermiform smooth appendix with a length of 8 cm and a diameter of 2 cm is present. An intraluminal cap of mucosa, 4* × *4* × *3 cm, is present at the base of the appendix and projects into the lumen of the caecum, 2 cm from the ileocaecal valve. Appendiceal lumen is smooth and contains thick mucus, no projections identified. Colonic mucosa has patchy areas of erosions.’*

**Fig. 4:**
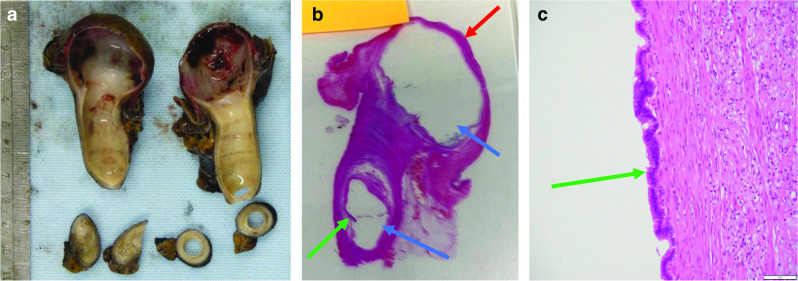
Macroscopic and microscopic histopathological tissue from proximal appendix: **a** illustrates the gross histological appendiceal specimen and **b** histological section showing a low-grade appendiceal mucinous neoplasm which involves the entire length of the appendix. The lesion undermines colonic mucosa at the caecal pole (red arrow) demonstrating ulceration and granulation tissue. Mucoid material can be seen extruding from the appendix (blue arrows), and mucinous goblet cells can be seen within low-grade neoplastic epithelium (green arrows) (**b**, **c**)

A 42-year-old male presented to the emergency department with crampy abdominal pain and bloody diarrhoea lasting 2 days, on a background of an 18-year history of UC. He reported multiple episodes of loose stools and fresh bleeding per rectum with one episode of vomiting. Regular medications included oral mesalazine 3 g once daily since diagnosis and subcutaneous adalimumab 40 mg bi-monthly for the 6 months prior. He initially attributed his symptoms to a ‘flare’ of UC, as a consequence of increased alcohol consumption of 70 units per week for the preceding 3 weeks. He did however note that this episode of abdominal pain was the most severe experienced to date. On physical examination, he was apyrexic, and haemodynamically stable. His abdomen was soft and non-tender. Bowel sounds were auscultated and normal. Laboratory findings were significant for leucocytosis of 20.5 × 10^9^/L and neutrophilia of 18.3 × 10^9^/L. C-reactive protein levels were 46 mg/L and haemoglobin was 14.8 g/gL. A colonoscopy was performed showing proctitis with spontaneous bleeding involving the distal 3–4 cm of the rectum. On visualisation of the left colon, the mucosa appeared normal. Upon inspecting the proximal transverse colon, a globular intraluminal mass was present (Fig. 1). At endoscopy, the performing gastroenterologist suspected a haemangioma or Meckel’s diverticulum that had intussuscepted. Dual contrast computed tomography (CT) of the abdomen and pelvis demonstrated a 12-cm fluid-filled ileocolic intussusception at the hepatic flexure with no definitive lead point (Fig. 2). There were no radiological findings concerning neoplastic pathology. Admitting surgeons reported this non-obstructing intussusception was unlikely to resolve through conservative management, so this patient proceeded to urgent exploratory laparotomy. At laparotomy, the intussusception was noted extending from the ileocecal region to the hepatic flexure. When the intussusception was reduced, the lead point was a grossly abnormal, dilated, inflamed appendix. Right hemicolectomy and ileocecal anastomosis was successfully performed. Histology reported 8.6 × 2.2 cm, low-grade appendiceal mucinous neoplasm (LAMN) involving the entire length of the appendix, histopathological stage TisN0 (American Joint Committee on Cancer 8th edition) (Figs. 3, 4) [5]. Twenty-three lymph nodes resected were negative for metastatic disease. The patient’s clinical condition improved between days 1–5 postoperatively and was discharged home day 11 postoperatively with maintenance therapeutics held on discharge. At 12 weeks of follow-up this patient required reintroduction of mesalazine followed by adalimumab at up due to relapse of his UC.

## Discussion

This clinical case illustrates complex interwoven medical, oncological and surgical pathological processes propelling this patient’s emergency admission. This patient’s extensive history and bona fide diagnosis of UC raised suspicion for an exacerbation of his disease or perhaps a benign or malignant neoplasm, however this presentation was due to a rare form of intussusception secondary to appendiceal LAMN. This highlights the clinical importance of ‘thinking outside the box’ when such patients present to the emergency room with a short history of severe abdominal symptoms.

Intussusception is an exceptionally rare finding in adult populations, typically mimicking other abdominal pathologies [3]. In adult patients, intussusception lead points are typically pathological in 90% of cases, 65% of which are neoplastic in nature [6]. Our case highlights these characteristics, in such that an ambiguous presentation was caused by LAMN and incidentally resected carefully at laparotomy. A favourable clinical outcome was achieved due to admitting physicians performing urgent endoscopy and subsequent CT scanning, leaving little option but to proceed to surgery. LAMN itself is rare diagnosis, and the natural history of the disease involves possible progression to pseudomyxoma peritonei (PMP) through the physiological redistribution phenomenon if left unresected [7, 8]. In this case, operating surgeons performed a meticulous resection of the appendiceal mass intraoperatively and consequentially prevented iatrogenic dissemination into the peritoneum. A 2018 case report from Burchard and colleagues reported a unique case of appendicular induced intussusception in an adult patient with UC [9], which was described by the authors as the sole documented case in medical and surgical literature. In light of this, we propose our adult presentation of intussusception with LAMN leading in the setting of UC as the second case of this nature currently documented. Furthermore, our review of the literature failed to yield results linking biologic therapies to incidence of LAMN or PMP or indeed to the incidence of intussusception, although the authors acknowledge this case describes dual pathologies in the setting of UC and biological therapy. Previous studies describe the benefit of chemotherapy in the setting of PMP, however the role of immunological therapies in LAMN/PMP is less well studied, and perhaps these therapies may potentiate the disease, as previously described with non-melanoma skin carcinoma and lymphoma [10–12].

Initially, our patient had a favourable clinical response to biological therapy prior to relapsing 12 weeks post-discharge from hospital. Recent hypotheses suggest that appendiceal tissue plays a role in the inflammatory and pathological processes driving symptomatic UC. In introducing the *PASSION* study, Sahami et al. postulate the appendix as a defective barrier which may alter the colonic microbiome, allowing aberrant interaction between bacteria penetrating the mucosa with innate immune cells. This potentiates abnormal immunological responses, which is thought to drive the pathophysiology of UC [13]. Rachmilewitz and Mizoguchi both proposed cytokine production by appendiceal tissue is responsible for triggering CD4+ T-cells in an immunological cascade in distal portions of the colon, causing symptoms in UC [14, 15]. These theories suggest that the anatomical and immunochemical properties of the appendix are somewhat responsible for distal inflammatory symptoms, and this patient’s remission in the immediate post-operative period would moderately support this. However, the relapse of his symptoms after 12 weeks suggests the multifactorial nature of UC with involvement of immune processes outside of the appendix and proposes appendicectomy will not prove beneficial in controlling the symptoms associated with this patient’s UC. The authors acknowledge novel theories regarding immunological crosstalk between the appendix and colonic tissue in diseases processes like UC, however we wish to emphasise the clinical the value of urgent evaluation using laboratory, endoscopic and radiological investigations in emergency cases as described.

## Conclusion

Intussusception is a particularly rare phenomenon in adult populations, with incidental LAMN as a clinical lead point even rarer. This clinical conundrum highlights how a number of rare clinical pearls may be amalgamated into an acute surgical presentation and the requirement to ‘think outside the box’ in clinical practice

## Data Availability

All data generated during this case report are included in this published article.

## Abbreviations

UC: Ulcerative colitis
IBD: Inflammatory bowel disease
CT: Computed tomography
LAMN: Low-grade appendiceal mucinous neoplasia
PMP: Pseudomyxoma peritonei
